# Optimized Memory Allocation and Power Minimization for FPGA-Based Image Processing

**DOI:** 10.3390/jimaging5010007

**Published:** 2019-01-01

**Authors:** Paulo Garcia, Deepayan Bhowmik, Robert Stewart, Greg Michaelson, Andrew Wallace

**Affiliations:** 1 Department of Systems and Computer Engineering, Carleton University, Ottawa, ON K1S 5B6, Canada; 2 Div. of Computing Science and Mathematics, University of Stirling, Stirling FK9 4LA, UK; 3 School of Mathematical and Computer Sciences, Heriot Watt University, Edinburgh EH14 4AS, UK; 4 School of Engineering and Physical Sciences, Heriot Watt University, Edinburgh EH14 4AS, UK

**Keywords:** field programmable gate array (FPGA), memory, power, image processing, design

## Abstract

Memory is the biggest limiting factor to the widespread use of FPGAs for high-level image processing, which require complete frame(s) to be stored in situ. Since FPGAs have limited on-chip memory capabilities, efficient use of such resources is essential to meet performance, size and power constraints. In this paper, we investigate allocation of on-chip memory resources in order to minimize resource usage and power consumption, contributing to the realization of power-efficient high-level image processing fully contained on FPGAs. We propose methods for generating memory architectures, from both Hardware Description Languages and High Level Synthesis designs, which minimize memory usage and power consumption. Based on a formalization of on-chip memory configuration options and a power model, we demonstrate how our partitioning algorithms can outperform traditional strategies. Compared to commercial FPGA synthesis and High Level Synthesis tools, our results show that the proposed algorithms can result in up to 60% higher utilization efficiency, increasing the sizes and/or number of frames that can be accommodated, and reduce frame buffers’ dynamic power consumption by up to approximately 70%. In our experiments using Optical Flow and MeanShift Tracking, representative high-level algorithms, data show that partitioning algorithms can reduce total power by up to 25% and 30%, respectively, without impacting performance.

## 1. Introduction

Advances in Field Programmable Gate Array (FPGA) technology [1] have made them the de facto implementation platform for a variety of computer vision applications [2]. Several algorithms, e.g., stereo-matching [3], are not feasibly processed in real-time on conventional general purpose processors and are best suited to hardware implementation [4,5]. The absence of a sufficiently comprehensive, *one size fits all* hardware pipeline for the computer vision domain [6] motivates the use of FPGAs in a myriad of computer vision scenarios, especially in applications where processing should be performed in situ, such as in smart cameras [7], where FPGAs embed data acquisition, processing and communication subsystems. Adoption of FPGA technology by the computer vision community has accelerated during recent years thanks to the availability of High Level Synthesis (HLS) tools which enable FPGA design within established software design contexts.

However, since FPGAs have limited on-chip memory capabilities (e.g., approx. 6MB of on-chip memory on high end Virtex 7 FPGAs), external memory (i.e., DDR-RAM chips connected to the FPGA) is often used to accommodate frames [8,9]. This causes penalties on *performance* (latency is much higher for off-chip memory access) and perhaps more importantly, on *size* (two chips, FPGA and DDR, rather than just FPGA), *power* (DDR memories are power hungry [10]) and have associated monetary costs, hindering the adoption of FPGAs.

In this paper, we research allocation of on-chip memory resources in order to minimize resource usage and power consumption, contributing to the realization of power-efficient high-level image processing systems fully contained on FPGAs. We propose methods for generating on-chip memory architectures, applicable from both HLS and Hardware Description Languages (HDL) designs, which minimize FPGA memory resource usage and power consumption for image processing applications. Our approach does not exclude external memory access: rather, it is orthogonal to any memory hierarchy, and applicable to any instances of on-chip memory. Specifically, this paper offers the following contributions:

- A formal analysis of on-chip memory allocation schemes and associated memory usage for given frame sizes and possible on-chip memory configurations.
- Methods for selecting a memory configuration for optimized on-chip memory resource usage and balanced usage/power for a given frame size.
- A theoretical analysis of the effects on resource usage and power consumption of our partitioning methods.
- Empirical validation of resource usage, power and performance of the proposed methods, compared to a commercial HLS tool.

Our experiments show that on-chip memory dynamic power consumption can be reduced by up to approximately 70%; using representative high-level algorithms, this corresponds to a reduction of total power by up to 25% and 30%, respectively, without impacting performance. The remainder of this paper is organized as follows: Section 2 describes related work within FPGA memory systems architecture and design for image processing. In Section 3, we formally describe the research problem of power-size optimization, present a motivational example that highlights the limitations of standard HLS approaches, and present alternative partitioning methods. Section 4 describes our experimental methodology and experimental results, and Section 5 presents a thorough discussion of said results. Finally, Section 6 presents our concluding remarks.

Throughout this paper, we use the term BRAM (Block Random Access Memory), a Xilinx nomenclature for on-chip memories, to refer to on-chip FPGA memories in general.

## 2. Background and Related Work

Within FPGA processing sub-systems, algorithms evolve from typical software-suitable representations into more hardware-friendly ones [6,11] which can fully exploit data parallelism [11] through application-specific hardware architectures [3], often substantially different from the traditional Von Neumann model, such as dataflow [12,13] or biologically inspired processing [14]. These heterogeneous architectures are customized for FPGA implementation not just for performance (e.g., by exploiting binary logarithmic arithmetic for efficient multiplication/division [15]), but also for power efficiency (e.g., by static/dynamic frequency scaling across parallel datapaths for reduced power consumption [16]).

More often than not, computer vision applications deployed on FPGAs are constrained by performance, power and real-time requirements [3]. Real time streaming applications (i.e., performing image processing on real-time video feeds [6]) require bounded acquisition, processing and communication times [16] which can only be achieved, while maintaining the required computational power, through exploitation of data parallelism [11] by dedicated functional blocks [7].

However, the greatest limiting factor to the widespread use of FPGAs for complex image processing applications is memory [9]. Algorithms that perform only point or local region operators (e.g., sliding window filters) [15] are relatively simple to implement using hardware structures such as line buffers [3]. However, complex algorithms based on global operations require complete frame(s) to be stored in situ [11]; examples of contemporary applications that require global operations are object detection, identification and tracking, critical to security. Notice we use the term “global operations” to simultaneously refer to two characteristics: the use of *global operators* (atomic operations which require the whole image, such as transposition or rotation) and *undetermined* (unpredictable) access patterns (e.g., a person identification system might only need a subset of a frame, but which subset cannot be decided at design time, as it depends on person location at runtime).

A possible approach is to refine image processing algorithms so they can perform on smaller frame sizes that can be contained on an FPGA [2]. Several algorithms maintain robustness for downscaled images [17], e.g., the Face Certainty Map [18]) or employ intelligent on-chip memory allocation schemes [8] to accommodate complete frames that take into account power profiles. The latter requires methods to optimize on-chip memory configurations in order to maximize valuable usage; often at odds with performance-oriented allocation schemes standard in HLS code generators. Other possible approaches include stream-processing algorithm refactoring to minimize memory requirements [19] or programming-language abstractions for efficient hardware pipeline generation [20]; these are orthogonal to our approach, and outside the scope of this work.

In our context, the most significant related work on the use of FPGA on-chip memory for image processing applications has focused on four aspects: processing-specific memory architectures, caching systems for off-chip memory access, partitioning algorithms for performance and on chip memory power reduction.

### 2.1. Processing-Specific Memory Architectures

Memory architectures specialized for specific processing pipelines typically exhibit poor BRAM utilization. Torres-Huitzil and Nuno-Maganda [9] presented a mirrored memory system: in order to cope with dual access required by computational datapaths; data is replicated in two parallel memories and a third one is used for intermediate computations. The need for data replication to support paralellism inhibits scaling for higher frame sizes. Mori et al. [21] described the use of neighbourhood loader: input pixels are fed to shift registers which de-serialize the input stream into a neighbourhood region. Their approach supports only one output port, and sequential region read (no random access). This approach does not exploit datapath parallelism, nor does it support classes of algorithms which require disparate region access. Chen et al. [22] use distributed data buffers for expediting Fast Fourier computations; they partially exploit spatial parallelism, focusing on time-multiplexing as a means for reducing resource-usage and power consumption. Although time-multiplexing is a convenient technique for certain classes of applications, it cannot be used in real-time streaming where input pixels arrive at steady rates (without discarding frames). Klaiber et al. [23] have developed a distributed memory that divides input frames into vertical regions stored in separate memories. Their approach allows fine grained parallelism, but is only capable of handling single-pass algorithms, i.e., which do not require storage of intermediate values. While this suffices for simple computations, it does not satisfy the requirements of sophisticated computer vision algorithms which process data iteratively (e.g., MeanShift Tracking [24]).

### 2.2. Caching Systems

Delegating frame storage to off-chip memory solves the capacity problem, at the cost of performance and monetary expense. Caching techniques are used to minimize the performance implications: e.g., Sahlbach et al. [25] use parallel matching arrays for accelerating computation; however, each array is only capable of holding one row of interest (the complete frame is stored in off-chip memory) and their results do not discriminate resource usage across modules, making it hard to estimate the precise array costs. This approach can only support a limited class of algorithms: column-wise operations, for instance, require off-chip memory re-ordering for data to be loaded on-chip as rows, consuming precious processing time. Similarly, Chou et al. [26] have shown the use of vector scratchpad memories for accelerating vector processing on FPGAs, but still rely on random-access external memories; a similar approach is followed by Naylor et al. [27] in the context of FPGAs as accelerators. The use of external memories solves the storage limitation: however, it greatly limits parallelism (only one access per external memory chip can be performed at once) or greatly exacerbates financial and power costs, if several external memories are used.

### 2.3. Partitioning Algorithms

For HLS-based designs, computer vision algorithms are naturally expressed by assuming frames are stored in unbounded address spaces [28]. This software approach to FPGA design not only easily exceeds FPGA memory capabilities but is also not easily integrated in streaming designs without significant refactoring. This has led to the development of custom hardware blocks and APIs for software integration [29]: “naive” C-based HLS results in several on-chip memory structures, whose sizes and interfaces are dependent on variables’ types, often sub-utilizing available on-chip memory. Most HLS tools offer compiler directives—*pragmas*—which guide the synthesis tool according to the designer’s intention: optimizing for performance through loop unrolling, or selecting different implementations (on-chip memories or LUTs). We advocate that more directives, invoking different synthesis strategies, are required in order to tackle design constraints such as space and power.

The majority of research into partitioning algorithms has mainly focused on performance: namely, throughput. Gallo et al. [30] have shown how to construct efficient parallel memory architectures through High-Level Synthesis: however, their approach is predicated on re-organizing memory placement at algorithm level, by examining computational behavior and placing data accordingly through lattice-based partitioning, which is not feasible on streaming applications where pixels are inputted sequentially. Although possible, it would require a complex memory addressing mechanism between pixel input and memory structure. The authors then expanded their work to incorporate information about loop unrolling [31], providing new partitioning algorithms for maximizing parallelism; however, they did not tackle the utilization problem. Similarly, Wang et al. [32] have demonstrated an extremely efficient algorithm for improving throughput, by creating memory structures that facilitate loop pipelining in high level synthesis. Their approach saves up to 21% of BRAMs compared to previous work [33]; still, since their objective is maximizing throughput, supporting loop pipelining, their approach does not achieve optimized memory allocation in terms of utilization efficiency.

### 2.4. Memory Power Reduction

The impact of memory partitioning on power consumption has been researched by Kadric et al. [34]. Their approach investigates the impact of parallelism, i.e., how data placement can be leveraged for parallel access, minimizing communication power. A similar approach is taken in [35]. Tessier et al. [36] show on chip memory power reduction through partitioning, similar to our approach and previous work by the same authors [37], and more recently in [38]. However, none of these investigations assume constraints on memory availability. In contrast, we investigate tradeoffs between power and scarce availability, inherent to the image processing domain, future work need clearly identified by Tessier et al: “an investigation to determine the optimal size and availability of different-sized embedded memory blocks is needed”.

## 3. Memory Partitioning on FPGA

In this paper we describe how to partition image frames into BRAMs in order to maximize utilization (i.e., minimize the number of required on-chip memories), subject to minimization of power consumption. We begin by by formulating the utilization efficiency problem, without paying any consideration to power aspects; the following section integrates power consumption in our problem formulation. We assume that only one possible BRAM configuration is used for each image frame buffer.

### 3.1. Problem Formulation: Utilization Efficiency

**Definition** **1.** *Given a BRAM storage capacity C, and a number of possible configurations i, the configurations set **Cfg** is a vector of i elements:*

(1) $$\mathrm{Cfg}=\left(\begin{array}{c} \left(M_{1},N_{1}\right)\\ \left(M_{2},N_{2}\right)\\ .\\ .\\ .\\ \left(M_{i},N_{i}\right) \end{array}\right)=\left(\begin{array}{c} Cfg_{1}\\ Cfg_{2}\\ .\\ .\\ .\\ Cfg_{i} \end{array}\right)$$

*where the first component of each element depicts BRAM width M and the second component depicts BRAM height N, such that:*

(2) $$M_{x}\times N_{x}\leq C,\forall x\in \left[0,i-1\right]$$

**Figure 1:**
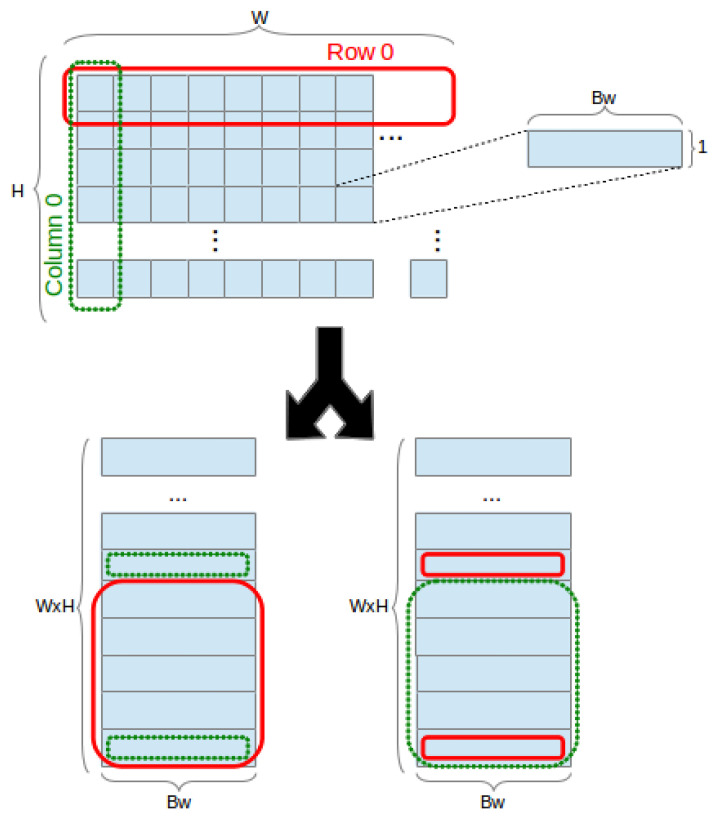
Mapping a 3-D array into row-major and colum-major order 2-D arrays.

For any given frame size, several possible BRAM topologies are possible (Different BRAM configurations do not always equal the same logical bit capacity. Whilst the total physical capacity is the same, in some configurations parity bits can be used as additional data bits. E.g., configuration (1,16384) can store 16384 bits, whilst configuration (9,2048) can store 18432 bits). A frame is a 3-dimensional array, of dimensions width *W*, height *H*, and pixel bit width $B_{w}$ (typically defined as a 2-dimensional array where the type defines the bit width dimension). BRAM topologies are defined based on a *mapping* of 3-D to 2-D arrays and a *partitioning* of a 2-D array to a particular memory structure (Figure 1).

Throughout the remainder of this paper, we assume the use of a mapping scheme which assigns $B_{w}$ to the *x* dimension and *H* and *W* to the *y* dimension, in both row-major and column-major order (where *x* and *y* are 2-D array width and height, respectively). This is the default approach in software implementations, where the type/bit width dimension is considered implicit, and a sensible approach for hardware implementations. Mapping bit width $B_{w}$ across the *y* dimension would result in implementations where different bits of the same array element (pixel) would be scattered among different memory positions of the same BRAM. This would require sequential logic to read/write a pixel, accessing several memory positions, creating performance, power and size overheads. It should be noted that this approach might offer performance advantages for certain classes of algorithms which might want to compare individual bits of different elements; however, we delegate this aspect to future work. Hence, we define only the default mapping scheme:

**Definition** **2.** *A mapping scheme m transforms a 3-D array **A3** into a 2-D array **A2** of dimensions x and y by assigning $B_{w}$ to the x dimension and ordered combinations of W and H to the y dimension, for a total of two possible configurations, as depicted in Figure 1. Mapping schemes are defined as:*

(3) $$\;\left(x,y\right)=m\left(W,H,B_{w}\right)$$

(4) $$\begin{array}{c} {A2}_{x,y}={A3}_{y\setminus W,y\%W,x},\;x=B_{w},y=W\times H \end{array}$$

(5) $$\begin{array}{c} {A2}_{x,y}={A3}_{y\%H,y\setminus H,x},\;x=B_{w},y=W\times H \end{array}$$

*where ∖ and % represent integer division and modulo, respectively.*

**Figure 2:**
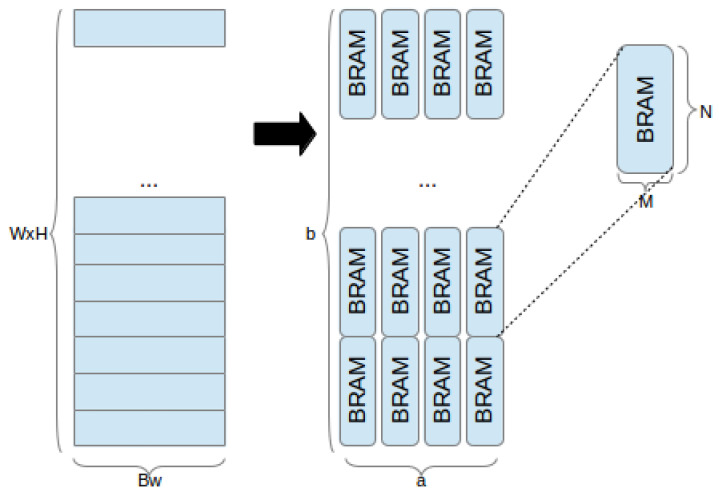
Mapping 2-D array of dimensions $x=B_{w}$ and y=W×H to a×b BRAMs configured for width *M* and height *N*.

**Definition** **3.** *Given a 2-D mapped image frame of dimensions x and y, a partitioning scheme p which assigns pixels across a × b BRAMs, depicted in Figure 2, is defined as the linear combination:*

(6) $$p\left(x,y\right)=\mathrm{Cfg}*\left(\begin{array}{c} \left(a_{1},b_{1}\right),\left(a_{2},b_{2}\right),\ldots ,\left(a_{i},b_{i}\right) \end{array}\right)$$

where * stands for linear combination, such that only one $\left(a_{x},b_{x}\right),\forall x\in \left[0,i-1\right]$ pair has non-zero components (such a pair is generated as a function of x and y), selecting $M_{p}$ and $N_{p}$ subject to:

(7) $$\left(\left(a\times M_{p}\right)\geq x\right)\cap \left(\left(b\times N_{p}\right)\geq y\right)$$

Different partitioning schemes *p*, implementing different functions of *x* and *y*, result in different addressing, input and output logic requirements, each with a particular impact on performance and resource usage. As this is the greatest bottleneck in implementing high-level image processing pipelines on an FPGA, it is paramount to define BRAM usage efficiency, i.e., the ratio between the total data capacity of the assigned BRAMs and the amount of data which is actually used.

**Definition** **4.** *Given a partitioning scheme p and maximum BRAM capacity C, the utilization efficiency E is defined as the ratio:*

(8) $$E=\frac{x\times y}{a_{p}\times b_{p}\times C}$$

The default mapping and partitioning schemes in state of the art HLS tools are geared towards minimizing addressing logic (abundant in contemporary FPGAs), resulting in sub-par efficiency in BRAMs usage (still scarce for the requirements of high-level image processing systems). Alternative schemes must be used in order to ensure memory availability within HLS design flows. We define the problem as:

**Problem** **1** (Utilization Efficiency)**.** *Given an image frame of width W, height H and pixel width $B_{w}$, select a partitioning scheme, in order to:*

*Maximize $E=\frac{x\times y}{a_{p}\times b_{p}\times C}$*

*Subject to $\left(\left(a\times M_{p}\right)\geq x\right)\cap \left(\left(b\times N_{p}\right)\geq y\right)$*

### 3.2. Utilization Example

Consider an image frame of width W=320 and height H=240, where each pixel is 8 bits (monochrome), and BRAMs which can be configured according to:

(9) $$\mathrm{Cfg}=\left(\begin{array}{c} \left(1,16384\right)\\ \left(2,8192\right)\\ \left(4,4096\right)\\ \left(9,2048\right)\\ \left(18,1024\right)\\ \left(36,512\right) \end{array}\right)$$

which is representative of state of the art FPGAs (Xilinx Virtex 7 family 18Kbits BRAM.), where total BRAM capacity *C* is given by C=36×512. Using a partitioning scheme

(10) $$p\left(m\left(320,240,8\right)\right)=\mathrm{Cfg}*{\left(\begin{array}{c} \left(8,8\right)\\ \left(0,0\right)\\ \left(0,0\right)\\ \left(0,0\right)\\ \left(0,0\right)\\ \left(0,0\right) \end{array}\right)}^{T}$$

where m(320,240,8)=(8,76800) (Equation (3)), yields a BRAM usage count of 64 (8×8 BRAMs configured for width 1 and height 16384), with storage efficiency:

(11) $$E=\frac{8\times (320\times 240)}{8\times 8\times (36\times 512)}=0.520833333$$

We have observed that this is the default behaviour for Xilinx Vivado HLS synthesis tools: empirical results show that configuration $\left(M_{1},N_{1}\right)=\left(1,16384\right)$ is selected through a partitioning scheme where $a_{1}=B_{w}$ and

(12) $$b_{1}=\frac{W\times H}{N_{1}}$$

rounded up to the nearest power of 2. Our experiments show that for any frame size, the synthesis tools’ default partitioning scheme can be given by:

(13) $$p\left(m\left(W,H,B_{w}\right)\right)=\mathrm{Cfg}*{\left(\begin{array}{c} \left(B_{w},2^{\left\lceil log_{2}\left(\frac{W\times H}{N_{1}}\right)\right\rceil }\right)\\ \left(0,0\right)\\ \left(0,0\right)\\ \left(0,0\right)\\ \left(0,0\right)\\ \left(0,0\right) \end{array}\right)}^{T}$$

where $2^{\left\lceil log_{2}\left(\frac{W\times H}{N_{1}}\right)\right\rceil }$ should be read as 2 to the *rounded up* (ceiled) result of the logarithm operation (i.e., 2 to an integer power).

Now consider the same mapping ($x=B_{w}$, y=W×H), but with a partitioning scheme:

(14) $$p\left(m\left(320,240,8\right)\right)=\mathrm{Cfg}*{\left(\begin{array}{c} \left(8,5\right)\\ \left(0,0\right)\\ \left(0,0\right)\\ \left(0,0\right)\\ \left(0,0\right)\\ \left(0,0\right) \end{array}\right)}^{T}$$

which partitions data unevenly across BRAMs, rather than evenly. This scheme yields a BRAM usage count of 40, with storage efficiency:

(15) $$E=\frac{320\times 240\times 8}{8\times 5\times (36\times 512)}=0.833333333$$

Yet a better partitioning scheme for the same mapping would be:

(16) $$p\left(m\left(320,240,8\right)\right)=\mathrm{Cfg}*{\left(\begin{array}{c} \left(0,0\right)\\ \left(0,0\right)\\ \left(2,19\right)\\ \left(0,0\right)\\ \left(0,0\right)\\ \left(0,0\right) \end{array}\right)}^{T}$$

yielding a BRAM count of 38 and efficiency:

(17) $$E=\frac{320\times 240\times 8}{2\times 19\times (36\times 512)}=0.877192982$$

Clearly, partitioning schemes depend on the frame dimensions, width, height, and bit width, to enable efficient use of on-chip memory blocks.

### 3.3. Power Considerations

Having formalized the utilization problem, we may proceed to analyse the power implications of each configuration. We model BRAM dynamic power consumption using the model described by Tessier et al. [37]: a power quantum is consumed per read and/or write. BRAM static power is directly proportional to utilization, hence addressed in the utilization problem.

For any given BRAM cell, the *read* power is consumed by a sequence of operations: the clock signal is strobed; the read address is decoded; the read data is strobed into a column multiplexer; the read data passes to BRAM external port. *Write* power is consumed by the following sequence: the clock signal is strobed; the write enable signal transfers write data to the write buffers; a line is selected by address decoding; data is stored in the RAM cell.

**Figure 3:**
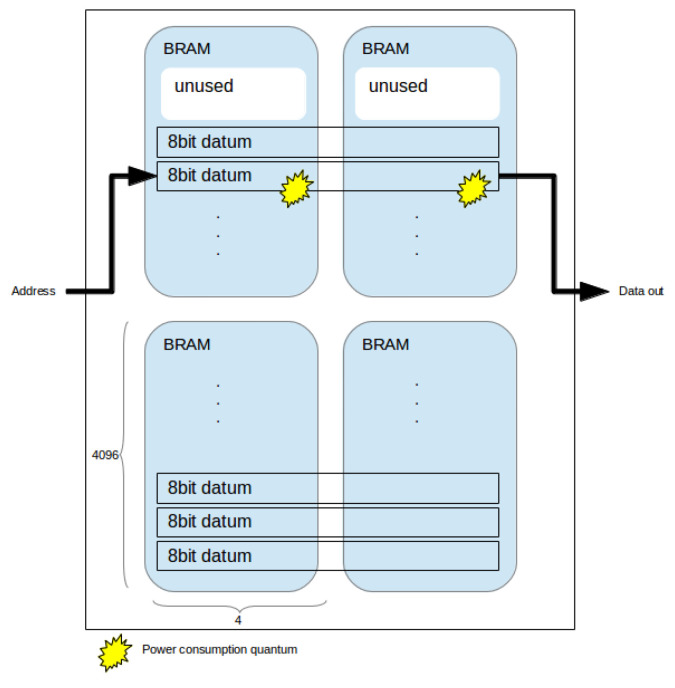
Partitioning across two BRAMs horizontally. Each access consumes two power consumption quantums.

**Figure 4:**
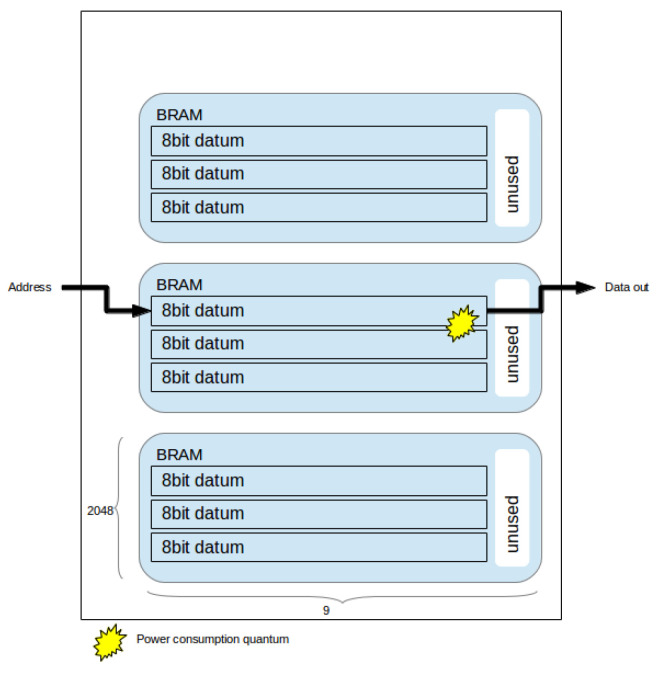
Partitioning across one BRAM horizontally. Each access consumes 1 power consumption quantum.

Now consider the partitioning presented in Equation (10) where each datum is distributed across eight BRAMs, and the partitioning presented in Equation (16), where each datum is distributed across two BRAMs. Each read/write operation in the former must consume power across four times the number of BRAMs in the latter. Figure 3 and Figure 4 depict examples of power consumption for two partitioning schemes.

A partitioning scheme which minimizes horizontal usage of BRAMs (i.e., across **x**) is more suitable for clock gating. Since fewer BRAMs must be accessed per operation, the proportian of unused ones, which can be effectively gated, increases. It is straightforward to implement clock gating through chip enable selection [39] which is enabled/disabled based on address decoding. colorred In other words, BRAM power consumption is proportional to the number of BRAMs required to access each pixel: and this number depends on which configuration is selected.

An intuitive approach to balance power consumption and utilization is to always use the widest BRAM configuration that suffices for $B_{w}$, or multiples of the widest available.

This, however, is not an optimized strategy. While it is true that dynamic power is reduced, static power might increase when moving from one configuration to a wider one since the total number of BRAMs might increase: utilization efficiency is modified. Additionally, the logic required for address (and chip enable) signals increases when moving to a wider configuration. This aspect makes the utilization and power problems indivisible. In the following section, we describe our approach to balance these two aspects.

### 3.4. Partitioning for Power and Utilization

We begin by presenting a brute force optimized partitioning procedure for maximizing utilization efficiency, described in Algorithm 1 in pseudo code notation.

**Algorithm 1** Optimized Utilization Efficiency can be achieved by:1: **procedure**
Optimized Partition 2:  efficiency←0 3:  best←0 4:  **for** x=0 : *i*-1 **do** 5:   $\left(\mathrm{Mx},\mathrm{Nx}\right)\leftarrow Cfg_{x}$ 6:   a←Bw/Mx 7:   b←W×H/Nx 8:   efficiency←(W×H×Bw)/(a×b×C) 9:   **if** efficiency **greater than** best **then** 10:    best←efficiency 11:    configuration←(Mx,Nx) 12:   **end if** 13:  **end for**

For each element in the configurations set **Cfg** (possessing a total of *i* elements), the procedure calculates the required number of BRAMs to store a frame of width *W*, height *H* and bit width *Bw*, the efficiency of such a configuration and compares it with the highest efficiency found so far. The focus here is solely on utilization. Effectively, this is an exhaustive search as the number of possible memory configurations is finite and this is an off-line process.

Table 1 depicts the configurations selected by procedure 1 for a representative number of frame sizes and pixel bit widths. Several of the configurations are not power-optimised: notice that for pixels of widths 10, 14 and 22, BRAM configuration 2 × 8192 is chosen most often (consuming power on 5, 7 and 11 BRAMs per access, respectively). This is intuitive from a utilization efficiency perspective: it is the only configuration that divides the width, and is in accordance with the selection of configuration 4 × 4096 for pixels of width 8, 12, 20 and 24 and configuration 18 × 1024 for pixels of width 18.

This non-linearity complicates the derivation of an optimized procedure for partitioning for both utilization and power efficiencies. Hence, we take a more relaxed approach and define a procedure through user defined *tradeoffs* (i.e., an estimation of how much BRAM utilization can be traded for power reduction) and power and space *heuristics*, based on empirical properties. Our brute force balanced method is described in Algorithm 2. It is assumed that the *tradeoff* is expressed in percentage points.

**Algorithm 2** Balanced Power-Utilization can be achieved by:1: **procedure**
Balanced Partition 2:  efficiency←0 3:  configuration←get_MxNx(OptimizedPartition()) 4:  best←get_efficiency(OptimizedPartition()) 5:  j←get_index(OptimizedPartition()) 6:  **for** x=j+1 : *i*-1 **do** efficiency 7:   $\left(\mathrm{Mx},\mathrm{Nx}\right)\leftarrow {\mathrm{Cfg}}_{x}$ 8:   a←Bw/Mx 9:   b←W×H/Nx 10:   efficiency←(W×H×Bw)/(a×b×C) 11:   **if** efficiency **less than** best - tradeoff **then** 12:    **break** 13:   **end if** 14:   configuration←(Mx,Nx) 15:  **end for**

Procedure 2 begins by selecting the optimized utilization solution and iterating over wider BRAM configurations (in the *x* dimension), calculating utilization efficiency. As long as the utilization is above the threshold limit, given by the difference between best utilization and tradeoff, in percentage points, the procedure continues. When it finds the first solution below the threshold, it exits, returning the last solution above the threshold limit. This approach follows the power model heuristics [37] described in the previous section: power consumption decreases as BRAM horizontal width increases (Figure 3 and Figure 4).

Table 2 depicts the BRAM configurations selected by the balanced procedure, with the tradeoff set to 12 percentage points. Compared to the optimized configurations, the majority of widths are increased, resulting in a more power efficient solution based on the aforementioned heuristics.

### 3.5. Applying Memory Partitioning: Methodology

**Figure 5:**
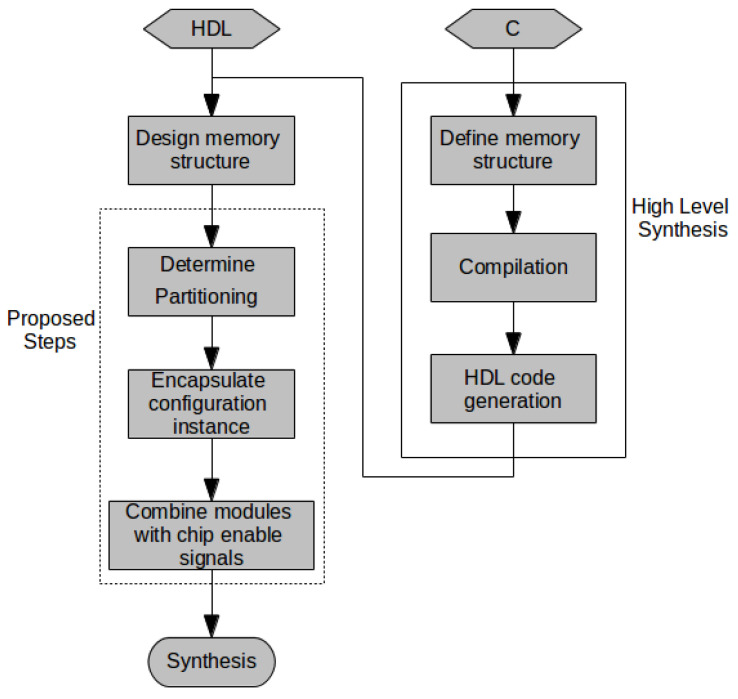
Proposed design flow from HDL and HLS, highlighting the additional steps required for minimizing utilization and power.

Our procedures can be utilized in both HDL and HLS design flows: in an HDL design flow, by guiding the designer’s implementation and/or refactoring; in an HLS design flow, through integration in the synthesis tools code generation subsystem. Figure 5 depicts the proposed design flows. The additional steps can be performed manually, either starting from HDL designs or by modifying HLS outputs pre-synthesis; through automated refactoring tools which compute the proposed procedures; or by the HLS tool prior to code generation. We describe the manual process used in our experiments.

After a memory structure has been derived from the procedure specification, according to Equations (3)–(5), procedures 1 and/or 2 are computed to determine BRAM partitioning. BRAMs of the computed configuration are instantiated and contained in modules (i.e., hardware entities). A top module instantiates all sub-modules, providing interfaces identical to the base HDL design or to the specification of the HLS tool. Addressing logic within the top module controls chip enable signals to each sub-module, ensuring that non-addressed BRAMs are not enabled. This careful partitioning of HDL logic in hierarchical modules, where addressing logic is determined by the top-level interconnect and BRAM configuration is determined by module configuration parameters ensures that the desired configurations are used (this is based on our experiments using Vivado: different synthesis tools might require additional compiler pragmas).

## 4. Experimental Results

Our experiments target state of the art FPGA devices (Xilinx Virtex 7 device xc7vx690tffg1761-1C and Zynq xc7z020clg484-1). We use Vivado v2016.1 for HDL design, Vivado HLS v2016.1 for High Level Synthesis, and Xilinx Power Estimator for power characterization of implemented designs. We begin by generating frame buffers in several configurations, in order to characterise utilization efficiency and power consumption. We then compare utilization and power against equivalent frame buffers generated by a HLS tool. We conclude by implementing two high level image processing algorithms through HLS, and modifying frame buffers according to the proposed strategies, in order to quantify our algorithms’ impact on resource usage and power consumption within complete image processing systems.

### 4.1. Frame Buffers: BRAM Configuration Impact

Our first set of experiments characterises utilization and power consumption for two frame sizes as a function of several possible configurations. The goal of this set of experiments was to validate the utilization efficiency of the partitioning algorithms and the power heuristics used in the previous section.

We implemented frame buffers in Verilog HDL in Vivado v2016.1, explicitly instantiating BRAMs according to the desired configurations. Logic in our design hierarchy routes data, addresses and control signals accordingly. Analysis of post-implementation reports was performed in order to ensure that BRAMs were instantiated according to the desired configuration (depending on the design hierarchy, synthesis tool optimizations could feasibly re-organize BRAM allocation). We performed a *sequential read/write* experiment, where a complete frame is written to memory (sequential pixel input, in row-major order) and then read in the same order. This allows us to validate the power model heuristics assumed in the previous section. Table 3 depicts power and utilization results for monochrome frames of sizes 320 × 240 and 512 × 512.

### 4.2. Frame Buffers: HLS Comparison

Our second set of experiments compares the proposed partitioning algorithms with default strategies employed by commercial HLS tools. The goal of this set of experiments was to confirm that the proposed methodology outperforms commercial HLS tools in both utilization and power consumption.

We performed C-based high level synthesis using Xilinx Vivado HLS, describing frames in the standard format (array type determines bit width, indices determine frame width and height). For each frame size, we report BRAM usage and additional resources (slice registers and LUTs). We utilized standard pixel widths (8 bits for monochrome images, 24 bits for RGB). We estimated optimized BRAM usage using the optimized utilization algorithm and according to the balanced partitioning algorithm in order to compare the power and utilization impact—algorithms were run offline; we have not integrated them in any HLS tool at this point. We implemented the frame buffers in Verilog HDL according to each algorithm, ensuring external interfaces (i.e., read/write data, address and control singals ports) are identical to the ones generated by Vivado HLS from C. We then replaced the frame buffers generated from HLS with our hand-coded Verilog HDL versions. For each frame size, we report BRAM usage and additional resources (slice registers and LUTs) required to implement addressing logic.

**Figure 6:**
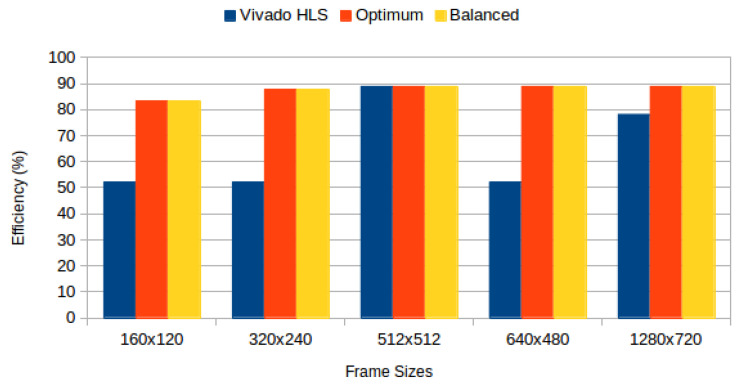
BRAM utilization efficiency for RGB frames: Vivado HLS versus proposed methods.

**Figure 7:**
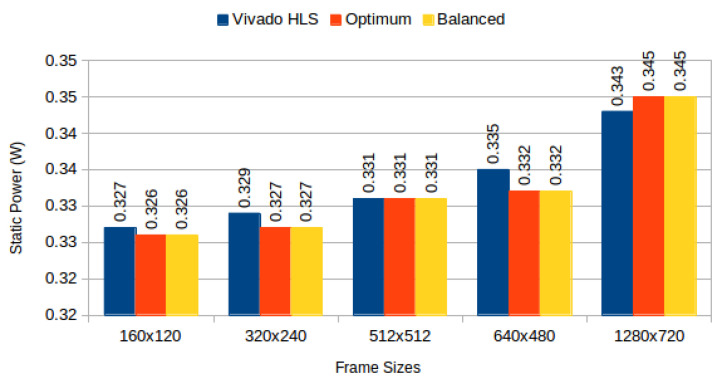
Static power consumption: Vivado HLS versus proposed methods.

**Figure 8:**
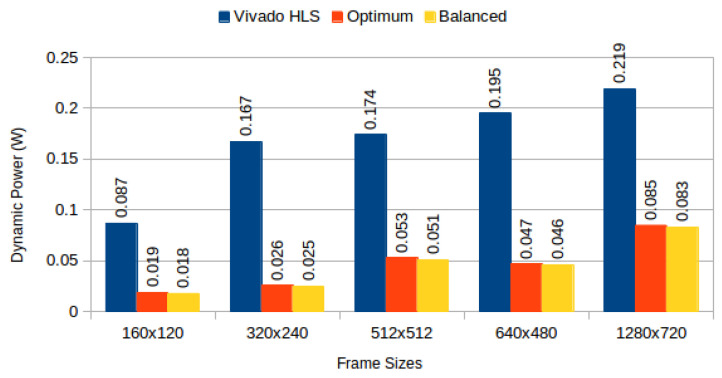
Total dynamic power consumption for sequential read/write: Vivado HLS versus proposed methods.

**Figure 9:**
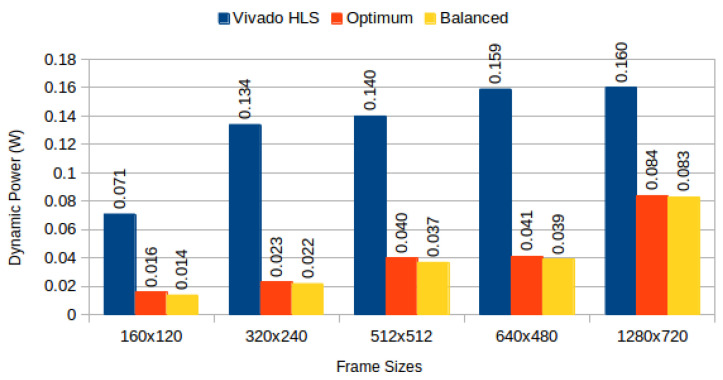
Total dynamic power consumption for 3 × 3 sliding window read: Vivado HLS versus proposed methods.

**Figure 10:**
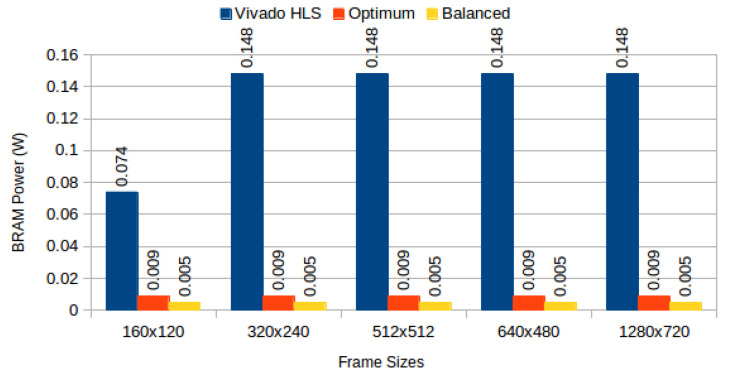
BRAM power consumption for sequential read/write: Vivado HLS versus proposed methods.

**Figure 11:**
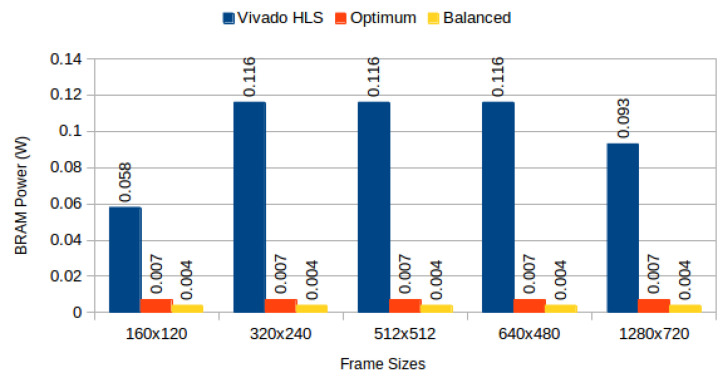
BRAM power consumption for 3 × 3 sliding window read: Vivado HLS versus proposed methods.

Table 4 depicts results obtained from the three configurations, for monochromatic and RGB frames respectively, and Figure 6 compares BRAM utilization efficiency. We characterised the power consumption implications of each generated system using Xilinx Power Estimator for access patterns representative of image processing applications. In our *sequential read/write* experiment, a complete frame is written to memory (sequential pixel input, in row-major order) and then read in the same order. In our *sliding window* experiment, a complete frame is read through 3 × 3 sliding window. Figure 7 depicts static power consumption; Figure 8 and Figure 9 depict total dynamic power consumption by the three architectures, for sequential read/write and sliding window test cases, respectively; and Figure 10 and Figure 11 depict BRAM power consumption for sequential read/write and sliding window test cases, respectively.

### 4.3. High-Level Image Processing

Out third set of experiments contextualises the impact of memory allocation on high-level image processing systems. The goal of this set of experiments was to quantify how much frame buffers impact resource usage and power consumption within complete image processing systems, based on default and proposed partitioning strategies.

**Figure 12:**
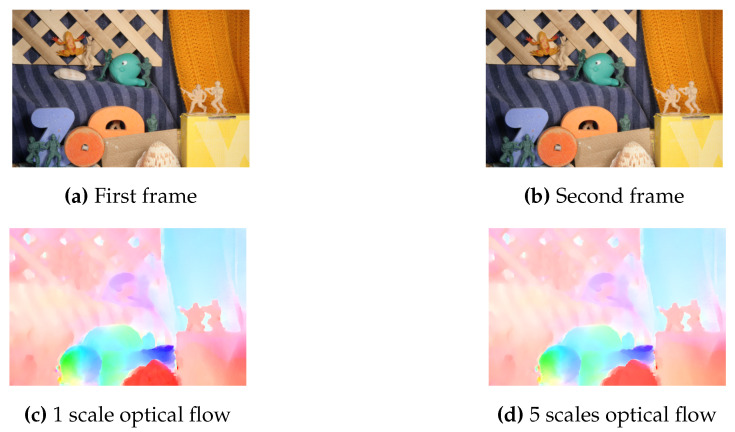
Optical Flow results using the implementation from [40]. (**a**,**b**): source frames. (**c**): output from 1 scale optical flow (used in our FPGA implementation). (**d**): output from 5 scales optical flow.

**Figure 13:**
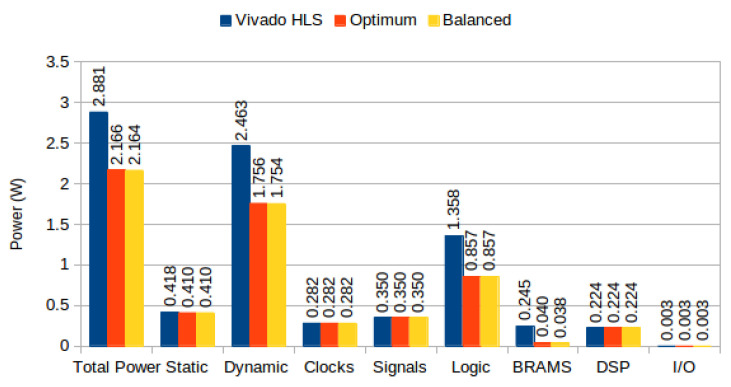
TV-L1 Optical Flow power consumption on Virtex 7.

We use Optical Flow and MeanShift Tracking as case studies. Optical Flow estimates the apparent motion of objects caused by the relative motion of an observer; i.e., for two sequential frames, Optical Flow estimates the movement of each pixel (or larger regions) from one frame to the other. It belongs to the *temporal* class of image processing algorithms, i.e., it performs computations across time (different frames). Our Optical Flow implementation is based on the code available from [40] using the TV-L1 method, refactored so it complies with Vivado HLS C synthesis requirements (e.g., dynamic memory allocation was replaced by static memory allocation); we performed no other optimizations. We compute a single scale, rather than multiple scales, for images of size 160 × 120: an example is depicted in Figure 12. We used the publicly available dataset from [41]. We developed three versions: with default memory allocation and following the optimized utilization and balanced algorithms. FPGA utilization results for Xilinx Virtex 7 are depicted in Table 5 (optimized and balanced strategies yield the same BRAM utilization, although different configurations, for our implementation). For the default strategy, BRAMs were insufficient to accommodate all memory requirements, causing the synthesis tool to infer Memory LUTs for parts of the design. Using our approach, BRAMs suffice to implement the complete system. Power consumption per version is depicted in Figure 13.

MeanShift Tracking [24] calculates a confidence map for object position on an image, based on a colour histogram of such object on a previous image: i.e., for an object whose position is known and colour histogram is calculated in frame *k*, MeanShift Tracking determines the most likely object position in frame *k + 1*, based on colour histogram comparison. It is a *temporal* and *dynamic* algorithm: it performs computations across more than one frame, requiring an unpredictable number of iterations (up to a predefined maximum) on unpredictable frame positions (depending on runtime object position). It was described in C and implemented through Vivado HLS; our implementation was highly optimized for hardware implementation. MeanShift Tracking stores the first input frame (writing the full frame to memory in sequential, row-major order) and calculates a color histogram of a region of width *M* and height *N*, centered on an initial object position (reading M×N pixels). Every subsequent frame is stored, and color histograms for possible new positions are calculated in a region around the previous known position. The new position is decided when the difference between previous and current position is below a pre-defined error bound or a maximum number of iterations is reached. The MeanShift tracking access patterns are not regular or predictable as they depend on the input images; it is representative of memory-intensive image processing algorithms as the output depends on complete (or unpredictable subsets of) scenes, rather than well-defined pixels or regions.

**Figure 14:**
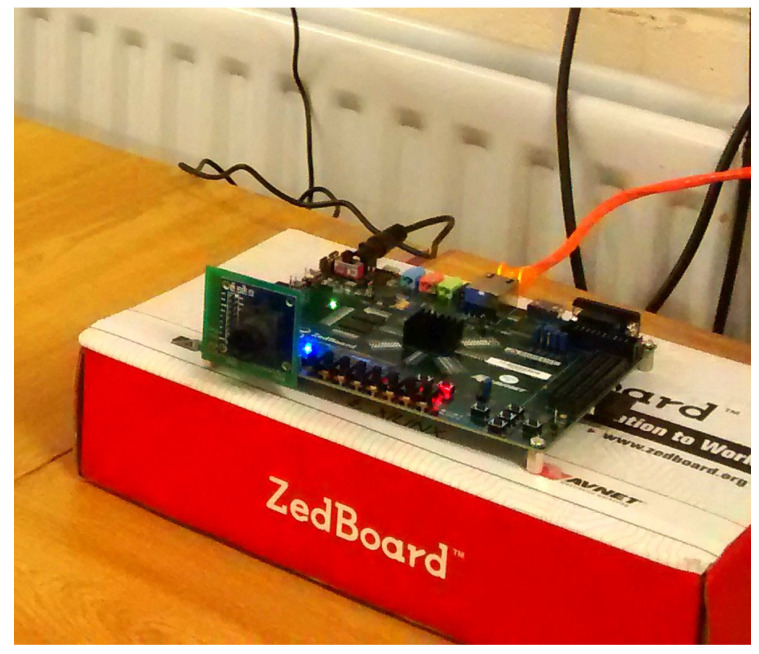
Zedboard connected to PC through Ethernet.

**Figure 15:**
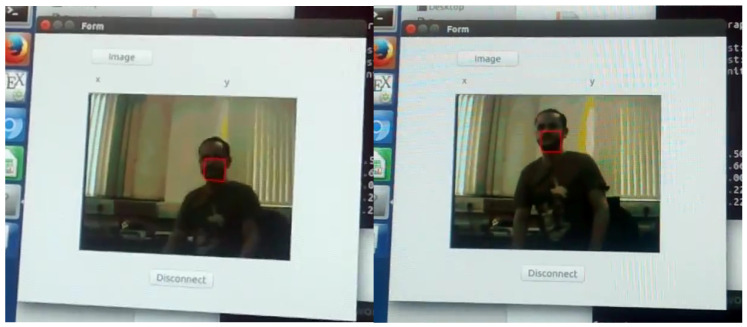
MeanShift Tracking: real-time face tracking displayed on PC. Image sent from Zedboard over Ethernet connection.

Our tracking system was implemented on a Zynq 7020 chip on a Zedboard, connected to an external camera OV7670 (Figure 14). The processed data (image plus tracked object position) are sent to the on-board ARM processor which re-transmits to a remote desktop computer over Ethernet. However, it is important to stress that this for communication and display only, the complete algorithm is implemented on the FPGA. Figure 15 shows real-time operation of our setup.

**Figure 16:**
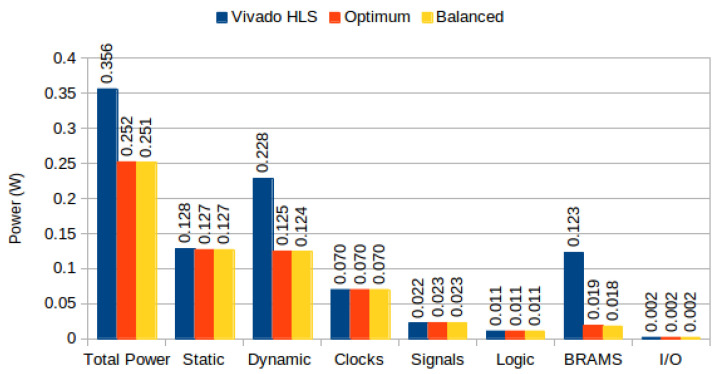
MeanShift Tracking power consumption on Zedboard.

We developed three system versions: with default memory allocation, optimized utilization memory allocation and balanced allocation for image sizes of 320 × 240 where each pixel is 24 bits (RGB), with a region of interest of size M=16 and N=21. Identical to the previous experiment, our baseline is the MeanShift Tracking implementation generated by Vivado HLS. The versions used for comparison replace the HLS frame buffer with hand-coded implementations: all other MeanShift Tracking modules are unmodified (generated from C through Vivado HLS). Resource usage for each version is depicted in Table 6. Power consumption per version is depicted in Figure 16.

## 5. Discussion of Results

Regarding the experiments in Section 4.1, we purposely chose these configurations in order to highlight the non-linear relationship between efficiency and power; while for frames of size 320 × 240, different configurations yield different efficiency and different power consumption, efficiency is identical across configurations for frames of size 512 × 512, while power consumption still varies. It is worthwhile noticing that for both sizes, BRAM configuration 9 × 2048 is less power efficient than configuration 4 × 4096, despite achieving the same efficiency; although BRAM power is decreased (from 0.009 W to 0.005 W in both cases), total dynamic power (comprised of BRAM, clocks, signals, logic and I/O) increases due to more complex logic, as previously described.

Experiments show that our partitioning algorithms achieve higher efficiency than default synthesis strategies, except for frames of size 512 × 512 where the efficiency is unchanged. This is the case where default strategies perform equally well in terms of utilization since the image height and width are powers of 2 (refer back to Equation (13)). This confirms that modified partitioning strategies are required, according to requirements, in order to improve memory usage.

Static power consumption depicted in Figure 7 decreases across frame sizes, except for frames of sizes 512 × 512 and 1280 × 720, where the utilization efficiency difference between default and proposed strategies is smallest (Figure 6) and additional addressing logic becomes too (static) power hungry. This confirms the utilization and power problems are indivisible, and must be treated in synergy.

Total dynamic power, on experiments performed on frame buffers, is reduced on average by 74.708% (σ= 7.819%) for read/write experiments (Figure 8), and on average by 72.206% (σ= 12.546%) for read-only experiments (Figure 9). This confirms our hypothesis that memory partitioning offers opportunities for power reduction, despite the need for logic overhead. Considering BRAM dynamic power only, our partitioning methods result in 95.945% average power reduction (σ= 1.351%) for read/write experiments (Figure 10) and 95.691% average power reduction (σ= 1.331%) for read-only experiments (Figure 11).

On our experiments using Optical Flow, where BRAM and Memory LUT power accounts for 25.9% of total power consumption, and 30% of dynamic power, we show that the proposed partitioning algorithms can reduce total power by approximately 25% (Figure 13). For MeanShift Tracking, where BRAM power accounts for 34.55% of total power consumption, and 53.94% of dynamic power, we show that the proposed partitioning algorithms can reduce total power by approximately 30% (Figure 16). Algorithm performance (i.e., frames per second) was unaffected by our partitioning methodologies, both in Optical Flow and MeanShift Tracking, since our strategies do not affect memory access latencies and maximum clock frequencies remained unchanged (frame buffers were not responsible for clock critical path). Our results compare favorably to the results presented in [36], which achieved up to 26% BRAM power reduction, at the expense of 1.6% clock frequency reduction; our methodology achieves up to 74% BRAM power reduction, without sacrificing clock frequency. This is due to the fact that their approach does not consider the power consumption differences caused by different BRAM configurations, a key aspect of our methodology.

### 5.1. Power Consumption

In Section 3.3, we illustrated how different BRAM configurations affect power consumption: depending on how many BRAMs must be strobed in order to access a pixel (in other words, depending on which configuration is used for memory allocation), different power consumption quanta are expended (assuming the remaining ones are clock gated, as per our methodology). The interested reader may refer to [37] for a detailed explanation of this power model. Figure 3 and Figure 4 visually display this phenomenon. Table 4 showed how, for the same frame size, different configurations can reduce power consumption expended on BRAMs by up to 82%; this corresponded to total dynamic power reduction of up to 50%. These results showed how severely BRAM configuration affects power consumption. Note that it is possible that a very small reduction in BRAM utilization (i.e., the number of BRAMs required to implement frame storage) can yield substantial power reductions.

In our experiments using complex high level algorithms, we showed that BRAM power constitutes a substantial portion of total power consumption: namely, using the default Vivado HLS strategy, BRAMs account for 8% of Optical Flow power consumption and 34% of Meanshift Tracking power consumption (Figure 13 and Figure 16). Additionally, significant power is spent on logic due to BRAM output change (prevented in our approach due to clock gating strategies).

### 5.2. Hardware Overhead

The default memory allocation strategy employed by HLS tools appears to be focused on minimizing addressing logic (implemented through LUTs), at the expense of memory usage. In contrast, our approach minimizes memory usage (a scarcer resource than LUTs) at the expense of more complex addressing. i.e., due to the use of different BRAM configurations, memory control logic (write-enable signals, address decoding, etc.) becomes slightly more complex, consuming more LUTs to implement. In our experiments using high level algorithms (Meanshift tracking and Optical Flow), this LUT overhead was of 0.8 and 2.0 percentage points, respectively (see Table 5 and Table 6).

## 6. Conclusions

Efficient mapping of high-level descriptions of image frames to low-level memory systems is an essential enabler for the widespread adoption of FPGAs as deployment platforms for high-level image processing applications. Partitioning algorithms are one of the design techniques which provide routes towards power-and-space efficient designs which can tackle contemporary application requirements.

Based on a formalization of BRAM configuration options and a memory power model, we have demonstrated how partitioning algorithms can outperform traditional strategies in the context of High Level Synthesis. Our data show that the proposed algorithms can result in up to 60% higher utilization efficiency, increasing the sizes and/or number of frames that can be accommodated on-chip, and reduce frame buffers dynamic power consumption by up to approximately 70%. In our experiments using Optical Flow and MeanShift Tracking, representative high-level image processing algorithms, data show that partitioning algorithms can reduce total power by up to 25% and 30%, respectively, without any performance degradation. Our strategies can be applied to any FPGA family and can easily scale as required for future FPGA platforms with novel on-chip memory capabilities and configurations.

The majority of HLS design techniques have focused on programmability and performance. However, our results show that further research is required in order to improve design strategies towards accommodating other constraints; namely, size and power. Models which describe low-level non-functional properties such as power consumption can support high-level constructs in order to display early cost estimation, guiding the design flow. This requires not only fine-grained characterization of technologies’ properties, but also sufficiently powerful modeling abstractions which can lift these properties to high-level descriptions. It will also be interesting to profile and refactor image processing algorithms to determine if alternative mappings (refer back to Equations (4) and (5)) could provide higher performance and utilization; this could be pursued in future work involving multi-objective optimizations.

Research in FPGA dynamic reconfiguration has focused on overcoming space limitations; whether this capability can be exploited for image processing power reduction, based on heuristics and runtime decisions, essentially transforming approximate computing design from a static to a dynamic paradigm, remains an open question.

## Figures and Tables

**Table 1:** BRAM configurations based on optimized utilization procedure.

	Pixel Width								
Frame	8	10	12	14	16	18	20	22	24
160 × 120	4 × 4096	4 × 4096	4 × 4096	18 × 1024	18 × 1024	18 × 1024	4 × 4096	9 × 2048	4 × 4096
320 × 240	4 × 4096	2 × 8192	4 × 4096	2 × 8192	18 × 1024	18 × 1024	4 × 4096	2 × 8192	4 × 4096
512 × 512	4 × 4096	2 × 8192	4 × 4096	2 × 8192	4 × 4096	18 × 1024	4 × 4096	2 × 8192	1 × 16384
640 × 480	4 × 4096	2 × 8192	4 × 4096	2 × 8192	18 × 1024	18 × 1024	4 × 4096	2 × 8192	4 × 4096
1280 × 720	4 × 4096	2 × 8192	4 × 4096	2 × 8192	18 × 1024	18 × 1024	4 × 4096	2 × 8192	4 × 4096

**Table 2:** BRAM configurations based on balanced procedure with tradeoff equal to twelve percentage points.

	Pixel Width								
Frame	8	10	12	14	16	18	20	22	24
160 × 120	9 × 2048	4 × 4096	4 × 4096	18 × 1024	18 × 1024	18 × 1024	4 × 4096	9 × 2048	9 × 2048
320 × 240	9 × 2048	4 × 4096	4 × 4096	18 × 1024	18 × 1024	18 × 1024	4 × 4096	9 × 2048	9 × 2048
512 × 512	9 × 2048	2 × 8192	4 × 4096	18 × 1024	18 × 1024	18 × 1024	4 × 4096	9 × 2048	9 × 2048
640 × 480	9 × 2048	2 × 8192	4 × 4096	18 × 1024	18 × 1024	18 × 1024	4 × 4096	9 × 2048	9 × 2048
1280 × 720	9 × 2048	2 × 8192	4 × 4096	18 × 1024	18 × 1024	18 × 1024	4 × 4096	9 × 2048	9 × 2048

**Table 3:** FPGA power usage and utilization efficiency (Eff.) for monochromatic (8 bits) frames of sizes 320 × 240 and 512 × 512 for different BRAM configurations.

320 × 240					512 × 512				
Configuration	Power (W)			Eff. (%)	Configuration	Power (W)			Eff. (%)
	Static	Dynamic	BRAM			Static	Dynamic	BRAM	
8 × 5–1 × 16384	0.328	0.054	0.036	83.33	8 × 16–1 × 16384	0.332	0.07	0.036	88.88
4 × 10–2 × 8192	0.327	0.036	0.018	83.33	4 × 32–2 × 8192	0.331	0.053	0.018	88.88
2 × 19–4 × 4096	0.327	0.026	0.009	87.72	2 × 64–4 × 4096	0.331	0.043	0.009	88.88
1 × 38–9 × 2048	0.327	0.027	0.005	87.72	1 × 128–9 × 2048	0.331	0.046	0.005	88.88

**Table 4:** FPGA resource usage for monochromatic frames: generated from Vivado HLS versus hand-coded modifications according to the proposed algorithms.

8 bits	HLS		Optimized Utilization				Balanced			
Frame	BRAMs	LUTs	BRAMs			LUTs	BRAMs			LUTs
			Usage	Mode	Reduction		Usage	Mode	Reduction	
160 × 120	16	0	10	4 × 4096	−37.5%	22	10	9 × 2048	−37.5%	48
320 × 240	64	9	38	4 × 4096	−40.6%	79	38	9 × 2048	−40.6%	186
512 × 512	128	17	128	4 × 4096	0%	285	128	9 × 2048	0%	596
640 × 480	256	34	150	4 × 4096	−41.4%	337	150	9 × 2048	−41.4%	742
1280 × 720	512	64	450	4 × 4096	−12.1%	1039	450	9 × 2048	−12.1%	2284
**24 bits**	**HLS**		**Optimized Utilization**				**Balanced**			
**Frame**	**BRAMs**	**LUTs**	**BRAMs**			**LUTs**	**BRAMs**			**LUTs**
			**Usage**	**Mode**	**Reduction**		**Usage**	**Mode**	**Reduction**	
160 × 120	48	0	30	4 × 4096	−37.5%	41	30	9 × 2048	−37.5%	91
320 × 240	192	25	114	4 × 4096	−40.6%	140	114	9 × 2048	−40.6%	308
512 × 512	384	49	384	1 × 16384	0%	504	384	9 × 2048	0%	1109
640 × 480	768	98	450	4 × 4096	−41.4%	584	450	9 × 2048	−41.4%	1285
1280 × 720	1536	192	1350	4 × 4096	−12.1%	1760	1350	9 × 2048	−12.1%	3877

**Table 5:** Optical Flow FPGA resource usage and performance on Virtex 7 xc7vx690tffg1761-1: generated from Vivado HLS versus hand-coded modifications according to the proposed algorithm.

	Vivado HLS Default	Optimized
FF	24101 (3%)	24101 (3%)
LUTs	200205 (47%)	208724 (49%)
Memory LUT	126114 (73%)	-
IOs	568 (67%)	568 (67%)
BRAM	1008 (35%)	2157 (74%)
DSPs	232 (7%)	232 (7%)
fps	24	24

**Table 6:** MeanShift Tracking FPGA resource usage and performance on Zynq 7020: generated from Vivado HLS versus hand-coded modifications according to the proposed algorithms.

	Vivado HLS Default	Optimized	Balanced
FF	6264 (5%)	6264 (5%)	6264 (5%)
LUTs	9197 (17%)	9310 (17.5%)	9475 (17.8%)
IOs	64 (32%)	64 (32%)	64 (32%)
BRAM	228 (81%)	150 (54%)	150 (54%)
DSPs	8 (3%)	8 (3%)	8 (3%)
fps	134	134	134
